# The relationship between hyperglycemia and the infection of COVID-19 in diabetic patients: A protocol for systematic review and meta-analysis

**DOI:** 10.1097/MD.0000000000021806

**Published:** 2020-09-04

**Authors:** Yan Liu, Yan Yang, Yalin Chen, Linyue Zhou, Qian Xiong, Chunguang Xie

**Affiliations:** a Hospital of Chengdu University of Traditional Chinese Medicine; b Chengdu University of Traditional Chinese Medicine, Chengdu, Sichuan Province, China.

**Keywords:** COVID-19, diabetes mellitus, Hyperglycemia, meta-analysis, protocol, systematic review

## Abstract

**Background:**

DM is a common chronic metabolic disease. COVID-19 is an infectious disease infected by enveloped single-stranded RNA coronavirus. Meanwhile, DM is a common comorbidity of SARS-CoV-2 infection. The virus can directly or indirectly damage the pancreatic islets and cause stress hyperglycemia by causing cytokine storms, acute inflammatory reactions, binding to the ACE2 receptor, etc. At the same time, hyperglycemia is a risk factor for severe infection and an independent risk factor for mild to severe disease. However, there is no evidence-based medicine to confirm the relationship between hyperglycemia and the infection of COVID-19 in diabetic patients. Therefore, we will conduct a systematic review and meta-analysis to synthesize the existing clinical evidence.

**Methods and analysis:**

We will retrieve each database from December 2019 to July 2020. Chinese literature comes from CNKI, Wanfang, VIP, CBM databases. English literature mainly searches Cochrane Library, PubMed, Web of Science, EMBASE. At the same time, we will look for clinical trial registration and gray literature. This study only included clinical randomized controlled trials. The reviewers independently conduct literature selection, data analysis, bias risk assessment, subgroup and sensitivity analysis. The primary outcomes include fasting blood glucose, 2-hour postprandial blood glucose, glycated hemoglobin, fasting insulin, adverse effects, etc. Finally, we will conduct a meta-analysis through Review Manager software version 5.3.

**Results:**

The results will be published in peer-reviewed journals.

**Conclusion:**

This study will explore the relationship between hyperglycemia and COVID-19 infection in diabetic patients. It will provide evidence-based support for clinical regulation of blood glucose and combating the COVID-19 epidemic.

**Registration number:**

INPLASY202060114

## 1 Introduction

Coronaviruses (CoVs) are enveloped single-stranded RNA virus.^[1]^ Novel coronavirus pneumonia (COVID-19) has a higher prevalence and lower mortality rate than Severe Acute Respiratory Syndrome Coronavirus (SARS-CoV). At the time of writing, COVID-19 has caused 10,420,325 infections and 508,467 deaths in the worldwide. Diabetes mellitus (DM) is a common chronic metabolic disease, accompanied by multiple organs damage and functions decrease. COVID-19 is generally susceptible around the world, however, diabetic patients often have multiple chronic diseases and a declining immune response, which is a high-risk group susceptible to infect by this virus. According to the current published literatures, the proportion of diabetes in COVID-19 patients is 10.1% to 20.0%, and the proportion of diabetes in critically ill COVID-19 patients is 22.2%.^[2–4]^

Many published documents indicate that after the body is infected with COVID-19, the virus will stimulate the patient's innate immune system and release a large amount of cytokines in the body, causing cytokine storms and acute inflammatory reactions, resulting in abnormal endothelial cell structure and function, impaired insulin delivery, and ultimately leading to insulin resistance and blood glucose high.^[5]^ Furthermore, according to a recent article published in the “Lancet” magazine, 43 out of 99 patients with COVID-19 developed liver damage that reduced the ability of liver cells to use glucose to synthesize glycogen, leading to increased insulin resistance and increased blood glucose.^[2]^ A study reported that Severe Acute Respiratory Syndrome Coronavirus 2 (SARS-CoV-2) can invade cells by binding to the receptor of angiotensin converting enzyme 2 (ACE2).^[6]^ Previous studies have found that in addition to human respiratory and lung tissues, ACE2 also expresses in pancreatic endocrine tissues.^[7]^ Therefore, it is speculated that the pancreas may also be the target organ for COVID-19 attack. The disease factor of elevated blood glucose in COVID -19 patients may be COVID-19 highly binding to ACE2 receptor in islet cells, causing islet cell damage.

However, the diabetic patients in a state of high blood glucose will cause excessive fat decomposition, decreased immunity,^[8]^ and prone to secondary infections, which in turn will lead to uncontrolled high blood glucose. In addition, long-term high blood sugar levels can cause oxidative stress, damage vascular endothelial cells, and affect all tissues and organs such as the heart, brain, and kidneys. Therefore, we must pay attention to the blood glucose management of patients with diabetes and covid-19, to help fight COVID-19 and diabetes.

Therefore, this article aims to explore the relationship between hyperglycemia and the infection of COVID-19 in diabetic patients. This result may help the government to formulate a more complete prevention strategy and provide new ideas for the clinical prevention and treatment of COVID-19 and diabetes.

## 2 Methods and analysis

### 2.1 Study registration

We have completed the registration of the systematic review protocol on the INPLASY website as INPLASY202060114 (https://inplasy.com/inplasy-2020-6-0114/). It is reported according to Cochrane Handbook for Systematic Reviews of Interventions, and the Preferred Reporting Items for Systematic Reviews and Meta-analysis Protocol (PRISMA),^[9]^ and the important protocol revisions will be recorded in the full review.

### 2.2 Inclusion and exclusion criteria

#### 2.2.1 Study design

Our research will include randomized controlled trials. However, repeated publications of the same study; articles on research in pediatric populations (17 years of age or younger); letters, abstracts, reviews, or animal experiments are excluded.

#### 2.2.2 Participants

We will include adult patients diagnosed with diabetes (using WHO 1999 diagnostic criteria^[10]^).

#### 2.2.3 Interventions and comparators

Diabetes patients diagnosed with COVID-19 are in the experimental group, while diabetes patients without COVID-19 are in the control group. Both groups receive conventional diabetes treatment recommended by the American Diabetes Association (ADA) guidelines,^[11]^ including diet, exercise, and hypoglycemic and lipid-lowering therapies. The experimental group receives conventional COVID-19 treatment recommended by the WHO guidelines, and the control group receives placebo or no treatment.

#### 2.2.4 Outcomes

The primary outcomes include fasting blood glucose, 2-hour postprandial blood glucose, glycated hemoglobin, fasting insulin, adverse effects, etc.

### 2.3 Study search

We will retrieve each database from December 2019 to July 2020. Chinese literature comes from CNKI, Wanfang, VIP, and CBM databases. English literature mainly searches Cochrane Library, PubMed, Web of Science, and EMBASE. We adopt the combination of heading terms and free words as search strategy, which decided by all the reviewers. Meanwhile, we will search other resources on the corresponding website to find the clinical trial registries and gray literature to complete the deficiencies of the electronic databases. Languages of the publications will be limited to English and Chinese. We will present the detailed search process in Table 1. Adjusting different search methods according to different Chinese and English databases.

**Table 1:**
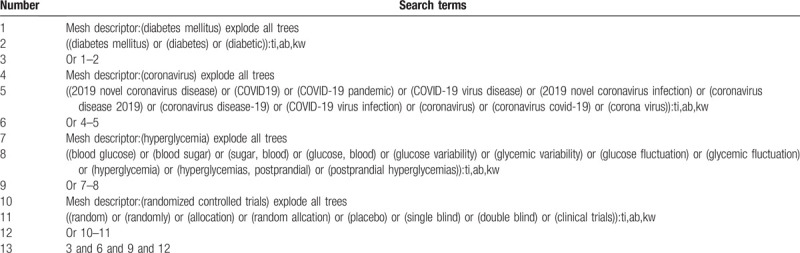
Example of Cochrane search strategy.

### 2.4 Data collection and analysis

#### 2.4.1 Selection of studies

**Figure 1:**
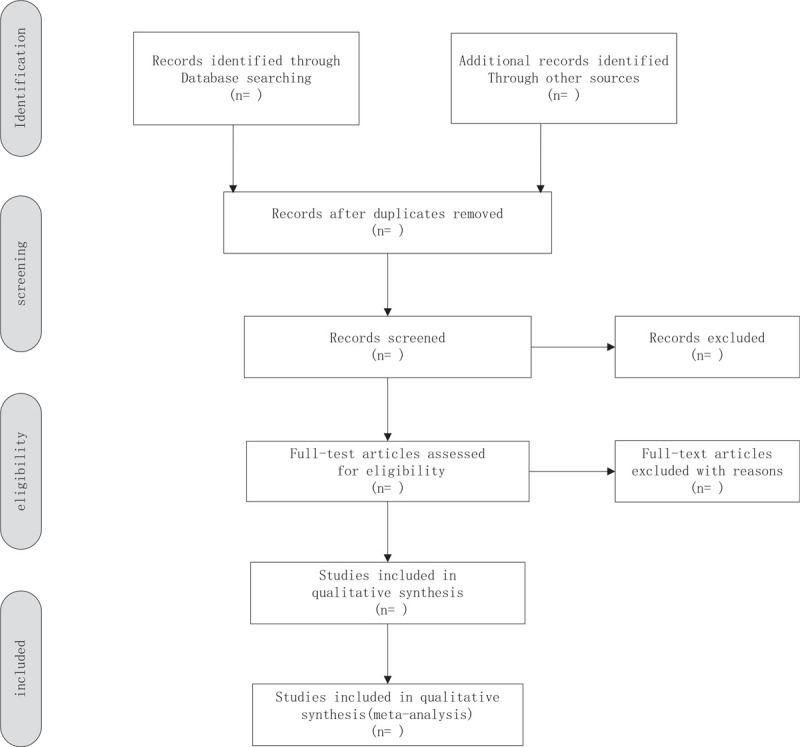
Flow chart of study selection.

Import all literatures that meet the requirements into the endnote X9 software. Two independent reviewers will conduct preliminary screening of the documents that do not meet the inclusion criteria of this study by reading the abstract and title, and then carefully read the full text to decide whether to include it. If an agreement cannot be reached in the above process, the agreement will be negotiated with the third parties. Besides, we will use a flowchart (Fig. 1) to show the identification and selection process of the study.

#### 2.4.2 Data extraction and management

Two independent reviewers will extract the qualified literature data into Microsoft Excel. We will extract the following information: title, author, year, sample size, age, gender, course of disease, intervention measures, outcomes and adverse reactions. If a key part of the article is missing, we will contact the corresponding author for complete information. If we are unable to get in touch with the author, we will exclude the study because of missing important information.

### 2.5 Risk of bias assessment

Two independent reviewers will evaluate the included literature on the risk of bias according to the guidelines of the Cochrane Handbook for Systematic Reviews of Interventions. The evaluation items are: random sequence generation, allocation concealment, blinding participants and personnel, blinding evaluation of results, Incomplete outcome data, selective result reporting, and other biases. The quality of each trial is divided into “low”, “high” or “unclear” risk of bias.^[12]^ When there are different opinions, the 2 reviewers can discuss or seek third-party consultation to reach a consistent conclusion.

### 2.6 Data analysis

We will use Review Manager software version 5.3 provided by Cochrane Collaboration to analyze the data. Dichotomous data is represented by 95% RR, and continuous data is represented by MD or SMD. When I^2^ <50%, *P* > .01 indicates that the study has no statistical heterogeneity, data will be synthesized using a fixed-effect model; on the contrary, when I^2^ ≥ 50%, *P* < .01, indicating the existence of considerable heterogeneity, the random effects model will be used for analysis.^[13]^ In addition, based on the different causes of heterogeneity, we will further conduct subgroup or sensitivity analysis to find potential causes. If meta-analysis cannot be performed, we will conduct a general descriptive analysis.

### 2.7 Subgroup analysis

We will divide the diabetic patients into the experimental group and the control group according to whether they have COVID-19 infection or not, and then conduct subgroup analysis based on different reasons such as age, gender, different forms of intervention, treatment process, drug dosage, etc.

### 2.8 Sensitivity analysis

In order to evaluate the robustness of the primary outcome measures, we will eliminate the low-quality studies and combine the data to assess the impact of the sample size, study quality, statistical methods and missing data on the meta-analysis results.

### 2.9 Publication bias assessment

If there are more than 10 studies in the meta-analysis, we will evaluate the symmetry of the funnel plot to examine the publication bias and interpret the results carefully.^[14,15]^

### 2.10 Grading the quality of evidence

The reviewers will assess the quality of evidence for the entire study in accordance with “grades of recommendations assessment, development, and evaluation (GRADE)” standard established by the WHO and international organizations. For simplicity and clarity, the GRADE system divides the quality of evidence into: “high,” “medium,” “low,” and “very low”. The GRADE profiler 3.2 will be employed for analysis.

### 2.11 Patient and public involvement

No patients and the public will participate in this study.

### 2.12 Ethics and dissemination

Since our research is a protocol for systematic review and meta-analysis, ethical approval is not required. Our research results will also be published in peer-reviewed journals.

## 3 Discussion

DM is a common chronic metabolic disease.^[16]^ With the influence of bad lifestyle, the disease has become the third largest non-communicable disease after cardiovascular diseases and tumors.^[17]^ COVID-19 is an infectious disease.^[18]^ Many clinical researches indicate that diabetes is a common comorbidity of SARS-CoV-2 infection. The virus can directly or indirectly damage the pancreatic islets and cause stress hyperglycemia by causing cytokine storms, acute inflammatory reactions, binding to the ACE2 receptor,^[19]^ etc. At the same time, hyperglycemia is a risk factor for severe infection and an independent risk factor for mild to severe disease.^[20]^ Therefore, special attention should be paid to diabetic patients among patients with COVID-19 infection.

However, there is no evidence-based medicine to confirm the relationship between hyperglycemia and the infection of COVID-19 in diabetic patients. Therefore, we attempt to conduct a meta-analysis to provide high-quality evidence for the regulation of blood glucose in diabetic patients with COVID-19 infections and provide new evidence for clinical response to the COVID-19 epidemic.

## Author contributions

The protocol was designed by YL, YY and YC under the guidance of CX. All the authors participated in the study. The manuscript was drafted by YL and revised by YY, YC and CX. All authors approved the final manuscript before submission. YL, YYand YC contributed equally to this work and should be regarded as co-first authors.

**Conceptualization:** Yan Liu, Yan Yang, Yalin Chen, Chunguang Xie.

**Data curation:** Yan Liu, Qian Xiong.

**Formal analysis:** Yan Liu, Yalin Chen.

**Investigation:** Yan Yang, Linyue Zhou.

**Methodology:** Yan Liu, Yan Yang.

**Project administration:** Chunguang Xie.

**Software:** Yalin Chen, Linyue Zhou.

**Visualization:** Yan Liu, Qian Xiong.

**Writing – original draft:** Yan Liu.

**Writing – review & editing:** Chunguang Xie.
